# Large Subcapsular Splenic Hematoma with a Large Pancreatic Pseudocyst Was Successfully Treated with Splenic Arterial Embolization and Ultrasound-Guided Percutaneous Drainage of Pancreatic Pseudocyst

**DOI:** 10.1155/2017/6381479

**Published:** 2017-08-22

**Authors:** Song Zhang, Fei Liu, Heena Buch, Guifang Xu, Lei Wang

**Affiliations:** Department of Gastroenterology, The Affiliated Drum Tower Hospital of Nanjing University Medical School, Nanjing, China

## Abstract

Subcapsular splenic hematoma is a rare complication of pancreatitis. The management for subcapsular splenic hematoma remains controversial. We herein report a case of a large subcapsular splenic hematoma with a large pancreatic pseudocyst, which was successfully treated with splenic arterial embolization and ultrasound- (US-) guided percutaneous drainage of pancreatic pseudocyst, for the first time. A 44-year-old male suffered from recurrent abdominal pain for more than two years. He had previous 3 episodes of pancreatitis. A subcapsular splenic hematoma (16.0 × 16.0 × 7.6 cm) with pancreatic pseudocyst (13.5 × 10.0 × 8.0 cm) was shown on abdominal computed tomography (CT). He underwent splenic arterial embolization to decrease the blood supply of the spleen and then ultrasound-guided percutaneous drainage of the large pancreatic pseudocyst. After 2 weeks, the repeated CT-Abdomen showed the disappearance of pancreatic pseudocyst and multiple areas of infarction on the spleen, while the splenic subcapsular hematoma had also significantly reduced. The patient was discharged after almost a month of his hospital admission with the drainage tube attached, and about 2 weeks later the drainage tube was removed upon CT scan confirmation of decrease in the volume of the subcapsular hematoma. Patient had no abdominal symptoms at the 1.5-year follow-up.

## 1. Introduction

A subcapsular splenic hematoma is a very rare hemorrhagic complication of acute or chronic pancreatitis. The prevalence of subcapsular hematoma of the spleen was estimated to be 0.4% in a recent study of 500 patients with chronic pancreatitis [1]. The management of this complication remains controversial. Thompson Jr. and Ashley [2] advocated early splenectomy to prevent rupture of the splenic hematoma and its associated morbidity. Rypens et al. [3] suggested that most of these complications could potentially regress and be managed conservatively. In addition, percutaneous drainage of the subcapsular splenic hematoma has been demonstrated in 4 case reports in the literature [4–7]. We herein present a case of a large subcapsular splenic hematoma with a large pancreatic pseudocyst successfully treated with splenic arterial embolization and US-guided percutaneous drainage of pancreatic pseudocyst, for the first time.

## 2. Case Report

A 44-year-old male was admitted to our hospital because of recurrent abdominal pain for more than two years that worsened over the past two weeks. He was a heavy drinker, with alcohol consumption of 250 g/day for 15 years. He had no history of trauma but previous 3 episodes of pancreatitis. He had the first episode of acute pancreatitis two years ago and received conservative treatment for it. The second episode of acute pancreatitis, about one and a half years ago, was accompanied by retroperitoneal cyst and splenomegaly along with dilation of the pancreatic duct. During the second episode, he underwent pancreatic duct dilation with a stent implanted into the pancreatic duct that was removed after 4 months. During the third episode of acute pancreatitis, about six months ago, a large pseudocyst was seen in the body and tail of the pancreas. Patient's symptoms were relieved after symptomatic treatment, but pseudocyst had not been treated. Nearly two weeks ago, the patient had recurrent upper abdominal pain, which was persistent and radiating to the left shoulder and back. He was admitted to local hospital. The routine blood test showed hemoglobin 102 g/L and serum amylase 139 U/L. Abdominal CT showed a large pseudocyst in the body and tail of the pancreas with signs of bleeding, a large subcapsular hematoma of the spleen, and cholecystitis with gallstones. Despite the conservative treatment consisting of pain control, bowel rest, intravenous fluids, and antibiotics, the pain was not relieved, so he visited our hospital.

**Figure 1:**
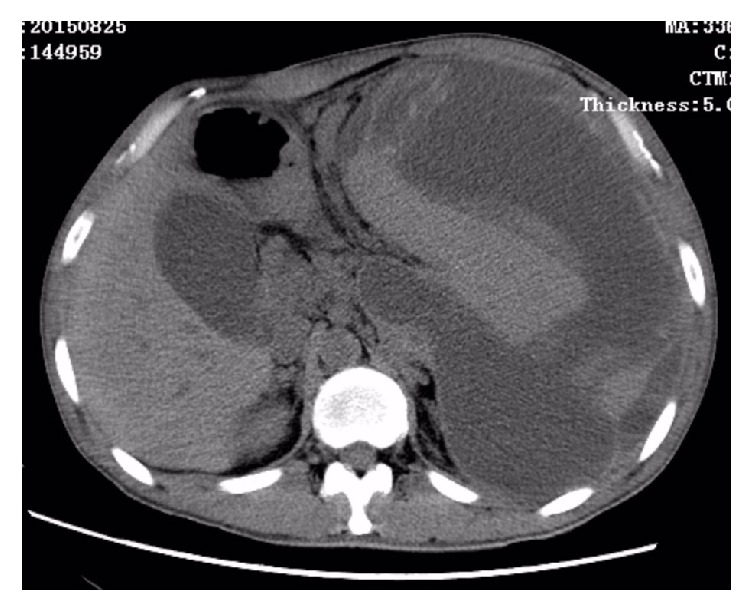
CT scan showing a large subcapsular splenic hematoma (16.0 × 16.0 × 7.6 cm) with a large pancreatic pseudocyst (13.5 × 10.0 × 8.0 cm).

**Figure 2:**
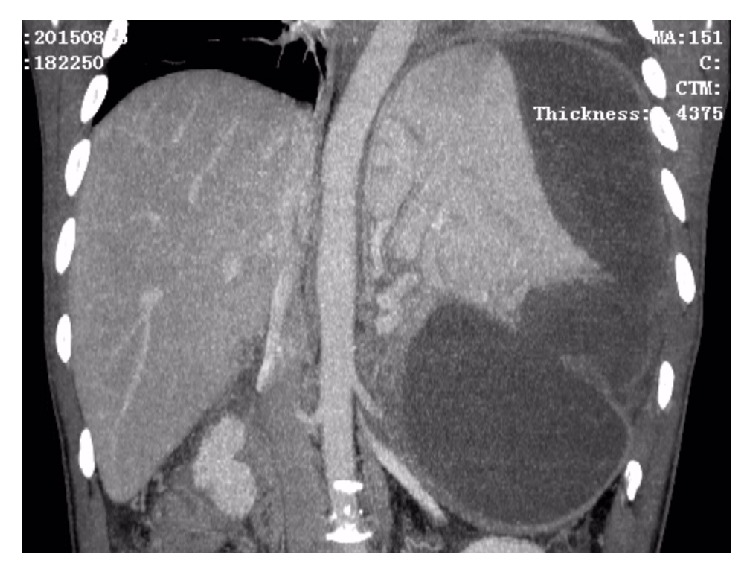
CT scan showing a large subcapsular splenic hematoma with a large pancreatic pseudocyst.

**Figure 3:**
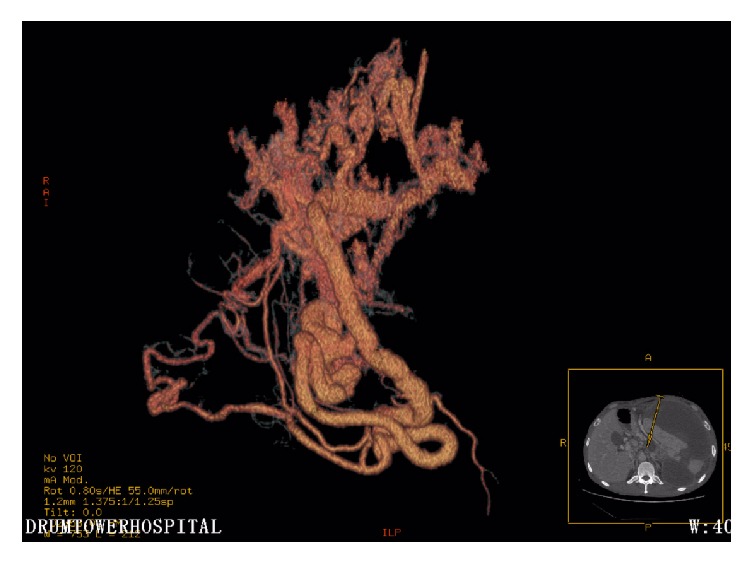
Vascular restructure with CT showing splenic vein dilation and expansion of portal vein branches.

After admission, his blood pressure, pulse rate, and body temperature were 117/69 mmHg, 58 beats per minute, and 36.5°C, respectively. The abdominal examination revealed a palpable mass in the left upper quadrant with mild tenderness. The spleen was palpable under the left costal arch. Murphy's sign was negative. The laboratory findings showed that the liver function tests, renal function tests, and electrolytes were within normal limit. However, there was a leukocytosis (12500/mm^3^), increased C-reactive protein (10.1 mg/dL), and decreased hemoglobin (9.1 g/dL) and hematocrit (26.9%). The serum amylase was 117 U/L, which were slightly above the normal range. The endoscopic ultrasonography revealed gastric vein varicosis in the fundus, pancreatic pseudocyst, cholecystitis with accumulation of biliary sludge, splenomegaly with subcapsular hematoma, and splenic vein varicosis. The CT-Abdomen revealed a huge low-density mass (16.0 × 16.0 × 7.6 cm) with some separations and loss of normal pancreatic morphology in the body and tail, splenomegaly with subcapsular hematoma (13.5 × 10.0 × 8.0 cm) that possibly pressurizes the left kidney, multiple cysts seen in the liver, gallstones, abdominal and pelvic effusion, bilateral pleural effusion with lungs atelectasis, splenic vein dilation, and expansion of portal vein branches (Figures 1–3).

**Figure 4:**
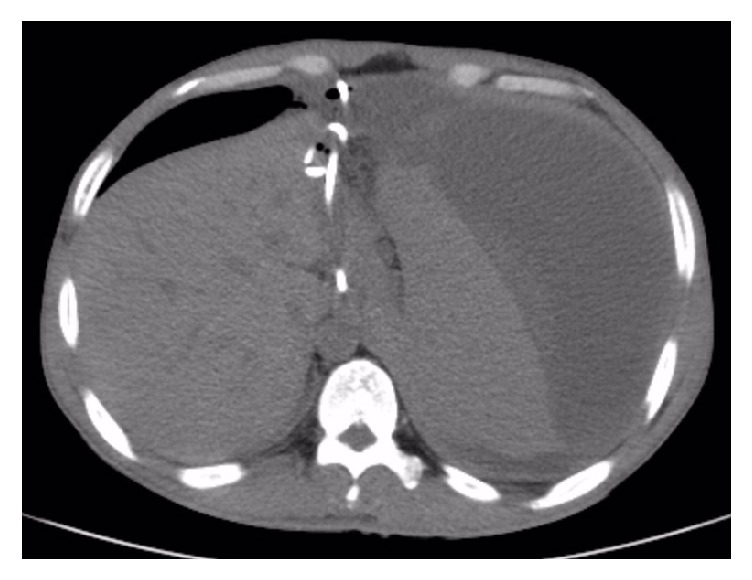
CT showing the drainage tube was in place, but splenic subcapsular hematoma had no change.

**Figure 5:**
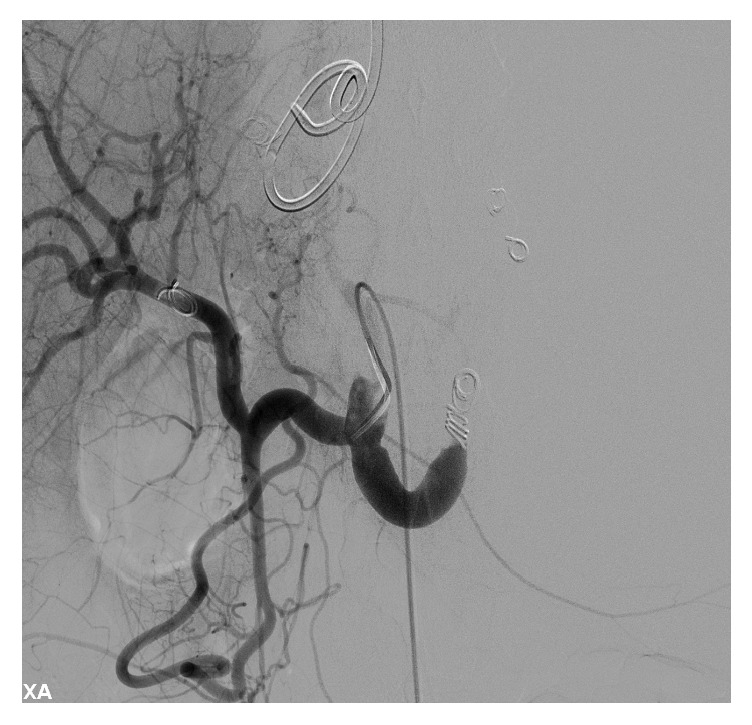
Showing coil embolization of splenic artery.

**Figure 6:**
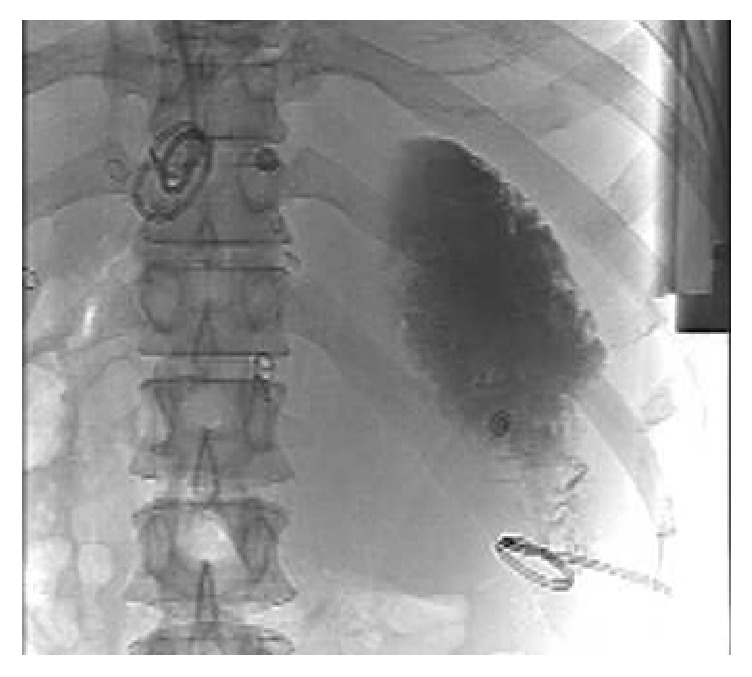
After US-guided percutaneous puncture drainage of pancreatic pseudocyst, X-ray showing drainage tube was in the right place.

**Figure 7:**
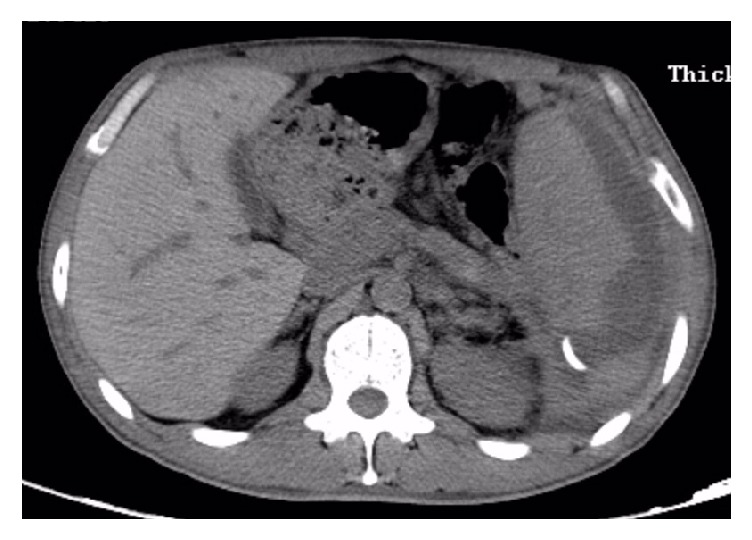
About 2 weeks after splenic artery embolization and ultrasound-guided percutaneous drainage of pancreatic pseudocyst, CT showing the disappearance of pancreatic pseudocyst, multiple areas of infarction on the spleen, and the significant reduction of splenic subcapsular hematoma.

**Figure 8:**
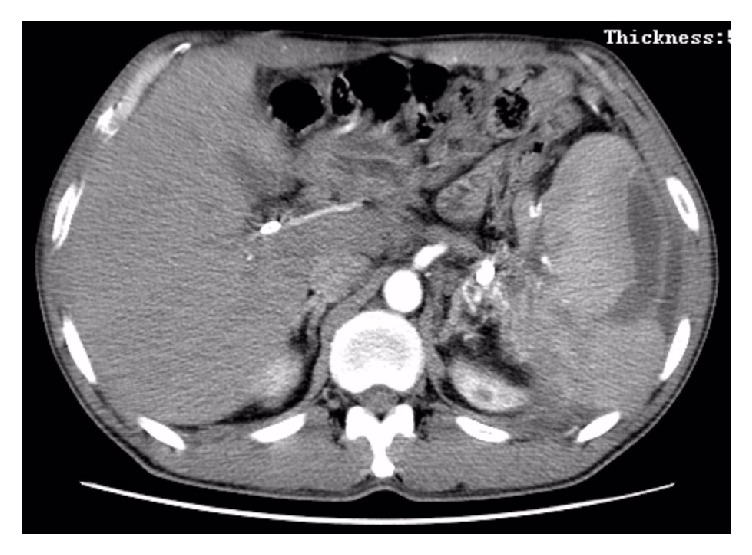
CT scan showing the volume of splenic subcapsular hematoma significantly reduced compared to all previous images.

During hospitalization, EUS-guided pseudocyst puncture and drainage were done, where small amount of dark brown liquid was collected. The following day CT-Abdomen showed no changes in the pancreatic pseudocyst and splenic subcapsular hematoma, while the NG tube was still in place (Figure 4). Splenic artery embolization (Figure 5) was done on the next day, with no significant changes on the follow-up CT-Abdomen result. About 4 days later, ultrasound-guided percutaneous puncture drainage of pancreatic pseudocyst was done (Figure 6). Postoperative fluid collection was about 5000 ml of dark brown liquid with amylase 21577 U/L, lipase 56704 U/L, red blood cell count 0.45 × 10^12^/L, and hemoglobin 29 g/L. The follow-up CT-Abdomen (plain + contrast), about 2 weeks later, showed the disappearance of pancreatic pseudocyst and multiple areas of infarction on the spleen, while the splenic subcapsular hematoma had significantly reduced (Figure 7). Abdominal ultrasound was done to obtain detailed status of the subcapsular hematoma. During hospitalization, the patient was also supplemented by antibiotics, nutritional support, acid suppression, and inhibition of pancreatic secretion. The patient was discharged after almost a month of his hospital admission with the drainage tube attached, and about 2 weeks later the drainage tube was removed upon CT scan confirmation of decrease in the volume of the subcapsular hematoma (Figure 8). Patient had no abdominal symptoms at the 1.5-year follow-up.

## 3. Discussion

In the present case, a large subcapsular splenic hematoma complicating large pancreatic pseudocyst gradually disappears after splenic arterial embolization and US-guided percutaneous drainage of a large pancreatic pseudocyst, and drainage of a subcapsular splenic hematoma and surgical procedures such as a splenectomy and distal pancreatectomy were avoided.

Splenic hematoma is a rare complication of acute or chronic pancreatitis when compared with traumatic origin of subcapsular hematoma. The tail of the pancreas is in close proximity to the splenic hilum, whereas the peritoneum encases the anterior pancreatic surface along with the splenic vessels [8, 9]. This anatomy provides a potential for splenic involvement in pancreatitis. There are several possible theories which might explain the mechanisms that how pancreatitis leads to subcapsular splenic hematoma: (1) erosion of splenic parenchyma after pancreatic enzyme leakage, resulting in either a splenic hematoma or intrasplenic bleeding, (2) disruption of splenic hilar vessels leading to subcapsular splenic hematoma, and (3) expansion of intrasplenic pseudocysts resulting in splenic rupture and bleeding [8]. In our case report, patient has a history of three episodes of pancreatitis. During the third episode, the CT scan showed pseudocysts with variable sizes at the body and tail of the pancreas, which were compressing the splenic vein and parenchyma. Therefore, according to the results of CT scan and enhancement imaging, we assume that direct erosion into the splenic parenchyma or disruption of the splenic vein might have led to a subcapsular splenic hematoma in this case.

Because splenic involvement in patients with pancreatitis is uncommon, the diagnosis of subcapsular hematoma of the spleen needs the alertness of physicians and imaging studies. Patients with pancreatitis exhibiting a mass in the left upper quadrant, pain radiating to the left shoulder, elevation of the left diaphragm, and a moderate fall in hematocrit should be suspected to have splenic complications. Imaging studies that are used to monitor the pancreatitis, such as CT and MRI, are regarded as reliable diagnostic tools and should be performed early in questionable patients. A splenic hematoma can be distinguished from simple fluid collection based on density (Hounsfield units > 30). Angiography is not essential for the diagnosis but would be indicated if splenic artery pseudoaneurysm, splenic vein thrombosis, or active bleeding was suspected [2, 6].

The management of subcapsular splenic hematoma in pancreatitis remains controversial, maybe due to its rareness. Some reports have advocated aggressive management with early splenectomy to avoid splenic rupture [1, 2, 10]. Thompson Jr. and Ashley [2] described 3 cases with acute pancreatitis accompanied by large subcapsular hematoma of the spleen. Two patients recovered after splenectomy, but 1 patient died due to continuing blood loss. They suggested that the treatment of subcapsular splenic hematoma should be a splenectomy in the vast majority of patients to prevent continuing blood loss and potential rupture. However, some authors advocate a conservative approach, which is based on the experience with traumatic lesions that can resolve spontaneously. Patel et al. [8] reported a case of subcapsular splenic hematoma managed conservatively with a favorable outcome, in which the CT scan showed the marked resolution of the hematoma after 4 months. This approach was considered as a safe management in hemodynamically stable patients. As a part of conservative approaches, percutaneous drainage of the hematoma, which showed good prognosis, may be increasingly used in recent years. Four cases of a subcapsular splenic hematoma have been reported which were successfully treated by percutaneous drainage of the hematoma [4–7]. Prompt relief of symptoms, a short recovery time, avoidance of rupture, and spleen preservation are the benefits of percutaneous drainage. In addition, Purushothaman and Borowski [11] also demonstrated the satisfying effect of splenic artery embolization for subcapsular splenic hematoma. However, it was just one particular case, which was reported.

In this special case, the patient suffered from a large subcapsular splenic hematoma with a large pancreatic pseudocyst. After repeatedly discussing about patient history and imaging studies in the multidisciplinary team (MDT), we considered that the only drainage of large pancreatic pseudocyst or spleen subcapsular hematoma will destroy the balance of the existing abdominal pressure, which may worsen subcapsular splenic hematoma, and the rupture of splenic hematoma and other unpredictable life-threatening consequences might even occur. Therefore, we made splenic artery embolization to decrease the blood supply of the spleen. Following, we made the US-guided percutaneous drainage of the large pancreatic pseudocysts. The postoperative abdomen CT scan demonstrated the significant resolution of pancreatic pseudocyst and subcapsular splenic hematoma, with relief of symptoms. There are several possibilities which might explain that subcapsular splenic hematoma gradually disappeared after splenic artery embolization and percutaneous drainage of the large pancreatic pseudocysts: (1) splenic artery embolism reduces the blood supply of spleen, and spleen subcapsular bleeding stopped; (2) after adequate percutaneous drainage of the large pancreatic pseudocyst, the pressure of splenic hilum decreased and the return of splenic vein increased which accelerated the absorption of hematoma; (3) there was the common channel between subcapsular splenic hematoma and pancreatic pseudocyst, so subcapsular splenic hematoma was also being drained while draining the pancreatic pseudocyst.

In conclusion, the definitive management of a subcapsular splenic hematoma complicating pancreatic pseudocyst is not yet established. Surgical intervention may be one of the therapeutic options for hemodynamically unstable patients, but with high mortality rate. Imaging-guided percutaneous drainage and splenic artery embolization appeared to be another feasible option for subcapsular splenic hematomas to prevent splenic rupture and obviate splenectomy. For the case of subcapsular splenic hematoma with a large pancreatic pseudocyst, the splenic artery embolization and US-guided percutaneous drainage of large pancreatic pseudocyst may be a safe and effective management.
